# A Real Stretch: Mechanisms Behind Cell Elongation

**DOI:** 10.1371/journal.pbio.1001782

**Published:** 2014-02-04

**Authors:** Mary Hoff

**Affiliations:** Freelance Science Writer, Stillwater, Minnesota, United States of America

Does tightening your belt make you taller? You might be tempted to conclude so after learning the results of an intriguing study of how notochord cells elongate in embryos of a primitive sea creature.

**Figure:**
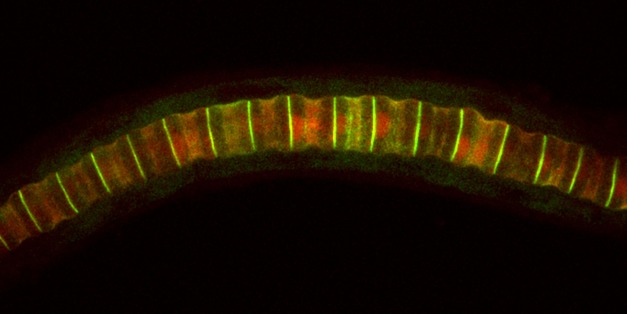
Interaction of various members of the actomyosin network is essential for the elongation of single notochord cells during the development of *Ciona intestinalis* embryos. *Image Credit: Dr. Ivonne M. Sehring*.

Embryonic development involves two basic processes: cell multiplication and cell shape changes. Elongation in particular is a crucial process for development of the notochord, which serves as a “backbone” for the developing animal and sets the stage for the organization of the rest of the body. It is also particularly intriguing: Elongation begins with the formation of a constrictive actomyosin ring around the middle of the coin-shaped cell, transforming it into an hourglass-shape and eventually into an elongated, drum-shaped cell. Is actomyosin's belt-tightening action responsible for notochord elongation? If so, how does it turn squeeze into stretch?

To shed light on the process, Di Jiang, Ivonne Sehring, Bo Dong, and colleagues took a close look at the formation, components, and activity of the actomyosin network that appears at the midsection of elongating notochord cells in the sea squirt *Ciona intestinalis*, which serves as a model chordate for development biology.

In other cells, the actomyosin ring is best known for being an integral part of the mechanism that splits one cell into two in the process of cell division, or cytokinesis. Along with actin and myosin, two proteins that work together in a ratchet-like formation to create force, the ring features a number of other proteins that help run the show, including actin-depolarizing factor/cofilin (which severs actin), tropomyosin (which appears to be involved in regulating the stability of actin), α-actinin (an actin regulator), and talin (which helps the actomyosin connect with the plasma membrane during cytokinesis).

The researchers first looked to confirm that the presence of the actomyosin ring was not due to cell division activity by looking for evidence of cell cycle processes, such as DNA replication. Finding none, they concluded that the equatorial constriction was not part of a cryptic cell cycle but was instead part of the cell elongation process.

Next, the team turned to elucidating the structure, formation, and function of the actomyosin ring. Using immunohistochemistry and fluorescent fusion protein analyses, they discovered that, as in the case of the actomyosin ring present in cell division, the area in which the constriction occurs is rich in cofilin, tropomyosin, α-actinin, and talin — all regulators of actomyosin ring contraction. Using time-lapse photography and other methods, they showed that cortical flow within the cytoplasm was responsible for the recruitment of both actin and myosin to the ring formation site. By creating mutants lacking various components of the ring infrastructure, they discovered that properly functioning cofilin and α-actinin are both needed for cell elongation. In other words, the structure and function of the notochord-lengthening actomyosin ring strongly resembles the ring responsible for cytokinesis, even though its job (lengthening the cell vs. splitting it in two) is very different.

Time-lapse photography of elongating notochord cells show frequent membrane deformations occurring at the basal surface during the elongation process, with a two-part cycle creating bleb-like formations that then retracted. Wondering what the deformation's connection might be to the constriction of the cell's midsection, the researchers looked at the molecular composition of the bleb. They discovered the presence of tropomyosin, cofilin, and the myosin-activating factor MRLC, suggesting that the retraction of the bleb-like deformation is due to individual contractions at the surface and not connected to the compression at the midsection of the cell. Further analysis revealed that as the bleb forms, the apical membrane moves toward the middle of the cell, then returns—suggesting that forces involved in basal cortex bleb retraction help generate the force needed to stretch the cell, contributing to the overall lengthening process.

Interestingly, the cells at either end of the notochord do not develop a waist or elongate. To determine whether that is a characteristic of the cell or of the location (having only one notochord cell neighbor), the researchers cut off the ends of the notochord. The new “end cell” also lacked the constriction and elongation function, confirming that having two notochord cell neighbors is key to the elongation process.

Putting it all together, the researchers concluded that notochord elongation in the model chordate they studied is due to a combination of equatorial constriction by an actomyosin ring that is essentially identical to the one that transforms one cell into two during cytokinesis, combined with local basal actin–driven contractions in the cytoplasm that help transform cellular belt-tightening into cellular stretch.

Finally, intrigued by the idea that an actomyosin complex known primarily for its cytokinetic function is also responsible for an entirely different job in non-dividing notochord cells, the researchers looked to other species in search of a more generalizable way of looking at the system's biological function. Close to home, they found a similar notochord-lengthening function in another tunicate, *Oikopleura dioica*. In addition, a literature search also revealed that actin-myosin complexes have been implicated in contraction functions in a diverse number of other species and organs, including fish eyes, amphibian nerves, plant roots, and fruit fly ovaries. The ubiquity of the structure and function, the authors concluded, suggests that such constriction is a widely used biophysical solution for lengthening biological units, from cells to entire embryos.

**Sehring IM, Dong B, Denker E, Bhattachan P, Deng W, et al. (2014) An Equatorial Contractile Mechanism Drives Cell Elongation but not Cell Division.** doi:10.1371/journal.pbio.1001781

